# Are men more likely to have serious occupational accidents? Findings of reported cases in Palmas, northern Brazil

**DOI:** 10.47626/1679-4435-2023-909

**Published:** 2023-04-18

**Authors:** Tiago Veloso Neves, Betania Moreira Cangussu Fonseca

**Affiliations:** 1 Centro de Referência em Saúde do Trabalhador, Secretaria Municipal de Saúde de Palmas, Palmas, TO, Brazil; 2 Curso de Bacharel em Medicina, Instituto Tocantinense Presidente Antônio Carlos, Palmas, TO, Brazil

**Keywords:** occupational accidents, occupational health, men’s health, acidentes de trabalho, saúde do trabalhador, saúde do homem

## Abstract

To ascertain whether male workers from Palmas, Tocantins state, northern Brazil, are more likely to have occupational accidents compared to female workers, all cases of serious occupational accidents reported between 2009 and 2019 were extracted from the Brazilian Notifiable Diseases Information System and compared with the economically active population according to sex. Men were found to be 6.2 times more likely to have a serious occupational accident compared to women. Therefore, reviewing occupational health and safety policies in predominantly male workplaces is necessary.

## Abstract (pt)

Para averiguar se trabalhadores do sexo masculino de Palmas, no estado do Tocantins, são mais propensos a terem acidentes de trabalho comparados com as do sexo feminino, foram extraídos do Sistema de Informação de Agravos de Notificação os casos de acidentes de trabalho graves notificados entre 2009 e 2019 e comparados com a população economicamente ativa de acordo com o sexo. Foi constatado que os homens apresentam 6,2 vezes mais chances de sofrerem um acidente de trabalho grave comparados com as mulheres. Portanto, é necessário rever as políticas de saúde e segurança do trabalhador nos ambientes de trabalho majoritariamente masculinos.

## INTRODUCTION

According to the Statistical Yearbook of Occupational Accidents,^1^ 440,914 cases of occupational accidents (OAs) were recorded in occupational accident reporting forms in Brazil in 2017. Of these, 294,810 cases (66%) involved men.

Not only do men predominate as OA victims, but they also seem to experience the worst outcomes. In the United States, 5,333 fatal occupational injuries were recorded in 2019, and 92% of deaths were among men.^2^

The predominance of male OA victims is a public health problem, as being affected by an OA may lead to temporary or permanent loss of income on which a family depends to survive. Several families still depend heavily on men’s financial contribution to their total income. Thus, this type of event may result in financial and social problems as well as suffering for the victim and/or family members.

Given that cases of serious occupational accidents (SOAs) are more frequent in male workers and may cause considerable harm, this study sought to ascertain whether men are more likely to have an SOA compared to women among the economically active population (EAP) of Palmas, capital of Tocantins state, northern Brazil.

## METHODS

This observational study was an ecological study, which is based on aggregated data. A software (Tab para Windows, TabWin) was used to extract SOA data reported in Palmas, from 2009 to 2019, and stored in the Brazilian Notifiable Diseases Information System (SINAN) database. These data were filtered according to the sex of the victims. Then, sex-specific numbers referring to the EAP of Palmas were collected from the Brazilian Institute of Geography and Statistics (IBGE) website, with 2010 population census data. A 2×2 contingency table was used to analyze the association between sex and OA occurrence. The number of accidents involving each sex was subtracted from the total number of economically active individuals of each sex, in an attempt to approach the comparison between having or not having had an OA.

The association between the two variables was analyzed using odds ratio (OR). The binomial test was used to ascertain whether the difference in the proportion of accidents between sexes was significant. P-values < 0.05 were considered significant for both the OR hypothesis test and the binomial test. Statistical analysis was performed in BioEstat 5.3.

This study is a development of the project “Agravos relacionados ao trabalho, incluindo COVID-19 relacionado ao trabalho, no município de Palmas, Tocantins: análise retrospectiva e prospectiva” [“Work-related injuries, including work-related COVID-19, in the municipality of Palmas, Tocantins: a retrospective and prospective analysis”, free translation], approved by the Research Ethics Committee at Fundação Escola de Saúde Pública de Palmas (CEP-FESP), with opinion number 4.677.414.

## RESULTS

According to IBGE (2010), the EAP of Palmas consists of 127,474 individuals, including 69,716 (54.7%) men and 57,758 (45.3%) women. Although the difference between sexes in the EAP is only 9.4%, there is a notable disparity between SOA victims reported between 2009 and 2019: 87.9% (3,542) were men. The difference in proportions of victims of both sexes within the EAP was considered to be of high statistical significance (p < 0.0001). Also, men were 6.2 times more likely to have an SOA than women, and the 95% confidence interval (95%CI) shows that this ratio varies very little in similar populations (95% CI = 5.72-6.92; p < 0.0001).

Table 1 shows the full EAP data, the number of SOAs by sex, and the OR, 95%CI, and p-values.

**Table 1:** Economically active population of Palmas and occupational accidents divided by sex

	Total	Men n (%)	Women n (%)
EAP	127,474 (100.0)	69,716 (54.7)	57,758 (45.3)
SOA	4,029 (100.0)	3,542 (87.9)	487 (12.1)
			
	OR	95%CI	p
	6.2	5.72-6.92	< 0.0001
			
Binomial test	z	p	Statistical power
	430.478	< 0.0001	1

Source: Brazilian Notifiable Diseases Information System (SINAN)/Brazilian Institute of Geography and Statistics (IBGE).

95%CI = 95% confidence interval; EAP = economically active population; OR = odds ratio; SOA = serious occupational accident.

The reported data from Palmas are consistent with the results of other studies, which found a male predominance in OAs in Brazil, in other American countries, and outside America. Thus, this seems to be a cross-cultural aspect.^1-3^ Men are believed to be more likely to take risks (in a broad sense)^4,5^ and, therefore, perform more dangerous jobs and accept working in more unsafe or unhealthy conditions. Therefore, any intervention aiming to reduce the number of accidents in this population should focus on organizational and environmental aspects that could minimize potential risks in work environments and processes. It is important to know that male workers, in general, may underestimate the consequences of these risks.

## CONCLUSIONS

Men are more likely to have an SOA compared to women. Although this disposition may be influenced by culture, the repeated pattern across different countries and cultures indicates a predominant biological influence that makes men more willing to take risks, which could be physical, financial, or of another nature. This characteristic behavior of male workers makes understanding and managing work environments and processes necessary in order to eliminate or mitigate the risks related to each occupation. If this group tends to take risks frequently, unhealthy and unsafe workplaces and work processes leading workers to exhaustion or not considering their characteristics and physiological needs will greatly increase the chances of SOAs among men.
